# Modulation of Intestinal Microbiome Prevents Intestinal Ischemic Injury

**DOI:** 10.3389/fphys.2017.01064

**Published:** 2017-12-19

**Authors:** Alessandra Bertacco, Carina A. Dehner, Giorgio Caturegli, Francesco D'Amico, Raffaella Morotti, Manuel I. Rodriguez, David C. Mulligan, Martin A. Kriegel, John P. Geibel

**Affiliations:** ^1^Department of Surgery, Yale School of Medicine, Yale University, New Haven, CT, United States; ^2^Hepatobiliary and Liver Transplant Unit, Università di Padova, Padova, Italy; ^3^Department of Immunology, Yale School of Medicine, Yale University, New Haven, CT, United States; ^4^Department of Pathology, Yale School of Medicine, Yale University, New Haven, CT, United States

**Keywords:** intestinal ischemia, intestinal transplantation, ischemia/reperfusion injury

## Abstract

**Background:** Butyrate protects against ischemic injury to the small intestine by reducing inflammation and maintaining the structure of the intestinal barrier, but is expensive, short-lived, and cannot be administered easily due to its odor. Lactate, both economical and more palatable, can be converted into butyrate by the intestinal microbiome. This study aimed to assess in a rat model whether lactate perfusion can also protect against intestinal ischemia.

**Materials and Methods:** Rat intestinal segments were loaded in an *in vitro* bowel perfusion device, and water absorption or secretion was assessed based on fluorescence of FITC-inulin, a fluorescent marker bound to a biologically inert sugar. Change in FITC concentration was used as a measure of ischemic injury, given the tendency of ischemic cells to retain water. Hematoxylin and eosin-stained sections at light level microscopy were examined to evaluate intestinal epithelium morphology. Comparisons between the data sets were paired Student *t*-tests or ANOVA with *p* < 0.05 performed on GraphPad.

**Results:** Lactate administration resulted in a protective effect against intestinal ischemia of similar magnitude to that observed with butyrate. Both exhibited approximately 1.5 times the secretion exhibited by control sections (*p* = 0.03). Perfusion with lactate and methoxyacetate, a specific inhibitor of lactate-butyrate conversion, abolished this effect (*p* = 0.09). Antibiotic treatment also eliminated this effect, rendering lactate-perfused sections similar to control sections (*p* = 0.72). Perfusion with butyrate and methoxyacetate did not eliminate the observed increased secretion, which indicates that ischemic protection was mediated by microbial conversion of lactate to butyrate (*p* = 0.71).

**Conclusions:** Lactate's protective effect against intestinal ischemia due to microbial conversion to butyrate suggests possible applications in the transplant setting for reducing ischemic injury and ameliorating intestinal preservation during transport.

## Introduction

Ischemia occurs when the blood supply to the small bowel is occluded, and if ischemia is followed by reperfusion, reoxygenation of the tissue occurs. During ischemia, an imbalance of metabolic demand and supply results in a hypoxic response with activation of hypoxia-inducible factor-1, apoptosis, autophagy, and necrosis (Eltzschig and Eckle, 2011). Paradoxically, the restoration of blood flow causes the release of proinflammatory molecules such as TNF-α, IL-6, and IL-1b, which exacerbate the injury. As a result, extra-intestinal organs, such as liver and the lung, may experience inflammatory activation and fatal multi-organ dysfunction syndrome.

In the clinic, intestinal ischemia and ischemia reperfusion injury (IRI) are commonly encountered in several clinical conditions including: atherosclerosis, hypotension, blood clots, hernias, cardiac and mesenteric surgery, venous thrombosis, necrotizing enterocolitis, trauma, shock, abdominal aortic surgery, and small bowel transplantation. Damage to the mucosal structure and barrier function due to IRI can lead to sepsis, systemic inflammatory response syndrome, and multiple organ dysfunction syndrome; consequently, the reduction of IRI is of considerable clinical importance.

Under normal conditions, the gastrointestinal tract provides resistance to both beneficial commensals and potentially pathogenic microorganisms. Perturbations to the intestinal microbiome, or dysbiosis, are correlated with numerous diseases, including inflammatory bowel disease (IBD) (Manichanh et al., 2006) diabetes (Wen et al., 2008), allergy (Penders et al., 2007), autoimmune diseases (Wu et al., 2010; Lee et al., 2011) and obesity (Ley et al., 2005). Dysbiosis can significantly affect the pathogenesis and severity of inflammatory diseases.

Short-chain fatty acids (SCFAs), and in particular butyrate, exhibit anti-inflammatory, antioxidant, and immunosuppressive effects. Since butyrate is the primary source of energy used in proliferating and differentiating colonic epithelial cells, it plays a key role in preserving the intestinal immune system and the mucosal barrier (Hill and Artis, 2010).

Recent studies have demonstrated in a rat model that butyrate pretreatment attenuates IRI by conserving epithelial structure and function (Qiao et al., 2015). Butyrate is the major product of lactate conversion by fecal microflorae (Bourriaud et al., 2005). In decreasing order, other SCFA produced include propionate, acetate, and valerate. Bifidobacteria's production of lactate has been demonstrated to be converted into butyrate by Eubacterium hallii (Duncan et al., 2004) and Anaerostipes caccae (Falony et al., 2006).

The odor of pure butyric acid is extremely pungent, making it problematic to tolerate as a therapeutic. Due to rapid absorption in the upper GI tract, colonic positive effects are reduced, which further limits its clinical applicability. Recently, however, a novel line of products that encapsulate butyrate inside a triglyceride matrix, leading to slower release in the GI tract, has been developed. Butyrate is also very expensive; for these reasons, we decided to use a butyrate precursor, namely lactate, to attempt to reduce IRI.

We hypothesized that the normal gut microbiome could locally protect against ischemia in the gut epithelium by metabolism of lactate to butyrate: here we present novel data using an intestinal perfusion unit (IPU) as well as histopathology to confirm this hypothesis.

## Materials and methods

### Bowel procurement

Male Sprague-Dawley rats (Charles River, Wilmington, MA) weighing 235–480 g, (weight-matched within experimental sets), were housed in climate and humidity- controlled, light-cycled rooms. After rats had been fully anesthetized by Isoflurane (IsoThesia TM, 99.9%/ml) (Dublin, OH), animal tissue dissection as performed in accordance with the guidelines of the Animal Care and Use Committee at Yale University (Protocol #2015-10253), as previously described in order to obtain the distal third of the small intestine was resected (average 20 cm) (Munoz-Abraham et al., 2015). The intestine was divided into two 10-cm segments and specified as either a control loop or an experimental loop. Both loops were perfused with HEPES solution (115 mM of NaCl, 5 mM of KCl, 1.2 of MgSO4, 1 mM of CaCl2, 10 mM of glucose, 2 mM of NaH2SO4, and 32.2 mM of HEPES at pH 7.4 and an osmolarity of 300 mOsm, at 37°C) to remove any remaining intestinal debris.

### *Ex-vivo* bowel perfusion device

Segments were attached to intestinal chambers of an *ex-vivo* bowel perfusion device (Munoz-Abraham et al., 2015). Control sections were perfused with 4 ml of HEPES containing 50 μM FITC-inulin (molecular weight 3,500 Da); experimental segments were additionally perfused with other agent(s), as indicated. FITC-inulin and lactate were purchased from Sigma Aldrich (St. Louis, MO). The extra-luminal sides of both intestines were circulated with HEPES. Both chambers were maintained in a 37°C water bath, and peristaltic 6 mL/min luminal flow was continuously provided.

### Experimental conditions

Four experiments perfusing with and without 1 mM lactate, and three experiments perfusing with and without 1 mM butyrate, were conducted under equal non-ischemic conditions.

Three ischemic trials perfusing with and without 1 mM lactate were conducted.

Ischemic trials were conducted by inducing ischemia in the control and experimental solutions by exposure to 100% N_2_ for 20 min before perfusion. In some experiments only the luminal solution was subjected to ischemia induction, while in others ischemia was induced in both the luminal and vascular solutions.

Six trials were conducted using 30-min pretreatment of 5 mM methoxyacetate inhibitor in order to interrupt the conversion of lactate into butyrate (Yamazoe et al., 2005). Here three trials perfused lactate with and without the inhibitor, while the other three trials perfused butyrate with and without the inhibitor.

Six antibiotic trials were conducted by prior luminal perfusion for 30 min of an antibiotic cocktail of 100 μg/mL Ampicillin, Metronidazole, and Neomycin and 50 μg/mL Vancomycin in HEPES solution. Trials were conducted with either vascular or luminal lactate (Table A1).

### Data acquisition

Absorption or secretion of fluid by the small intestine leads to an increase or decrease of FITC-inulin concentration, respectively. FITC, a fluorescent tracer fluorescein isothiocyanate, is attached to a biologically inert sugar inulin (FITC-Inulin), which when perfused through the lumen, allows for the evaluation of intestinal graft functional state. Identification of FITC-inulin in the bath shows a loss of integrity from lumen to bath.

A nanofluorospectrophotometer (Nanodrop 3300, Thermo Fisher Scientific Inc., Wilmington, DE) measured fluorescence intensity. Measurements were recorded from both ends of the bowel perfusion device to ensure proper perfusate flow. In addition, samples of the basolateral perfusate were also taken; if there was a signal detected in these samples the experiment was discarded, as this was indicative of leak or a perforation of the segment. Recorded measurements correspond to specific time points of concentration collected at 0, 15, 45, 60, and 90 min after perfusion began.

### Histopathology

After 30-min static infusion, 2 cm samples were obtained from the central portion of control and experimental sections, and fixed in 10% buffered paraformaldehyde for 24 h. Hematoxylin and eosin (H&E)-etched and stained 1 μm sections were obtained from Epon-embedded tissue for light level plastic sections. Intestinal damage was evaluated by a blinded pathologist according to the Park Chiu score (Table A2).

### Statistical analysis

Data values are expressed as the mean of the FITC concentrations of control vs. experimental sections. Comparisons between data sets were performed by paired Student *t*-tests or ANOVA (GraphPad Prism 6; GraphPad Software, La Jolla, CA), with *p* < 0.05.

## Results

### Prevention of ischemic injury by butyrate and lactate perfusion

**Figure 1:**
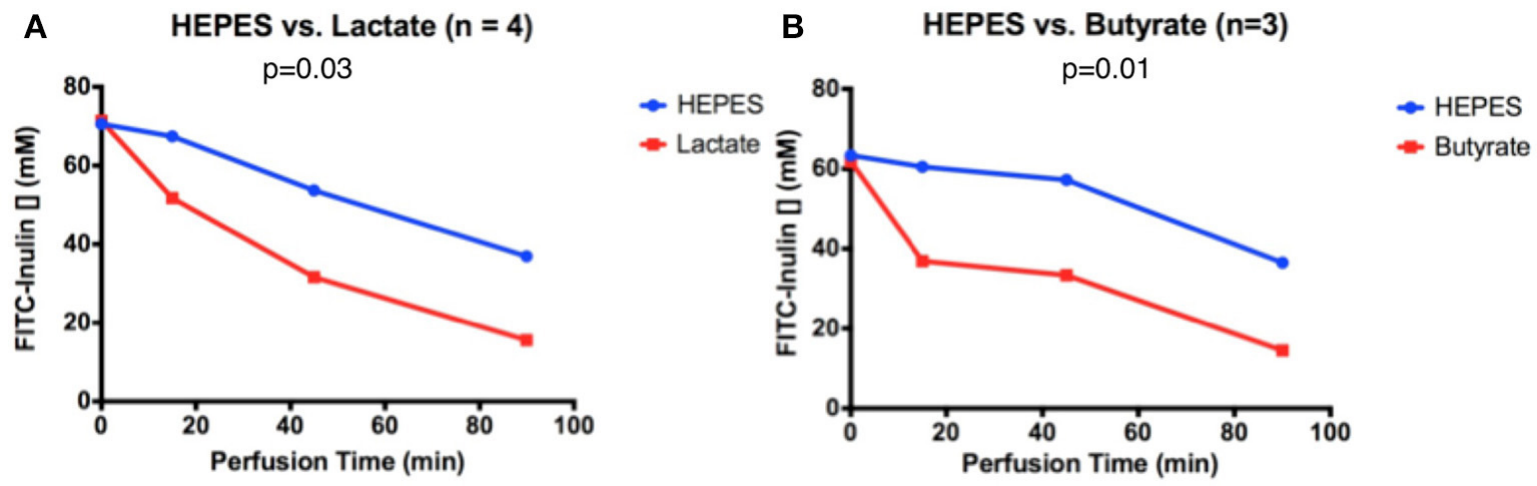
FITC-inulin concentration over 90 min in intestines perfused with HEPES and **(A)** lactate or **(B)** butyrate.

In the butyrate-perfused intestine, FITC-inulin concentration decreased from 62 to 15 μM over 90 min of perfusion. It exhibited a significantly greater and accelerated decrease in FITC-inulin concentration than the intestine perfused only with HEPES, with a final discrepancy of 22 μM at 90 min (*p* = 0.01, Figure 1B). In the lactate-perfused intestine, FITC-inulin concentration decreased from 70 to 20 μM over 90 min of perfusion. It exhibited a significantly greater and accelerated decrease in FITC concentration than the intestine perfused only with HEPES buffer, with a final discrepancy of 21 μM at 90 min (*p* = 0.03, Figure 1A).

### Amelioration of intestinal histopathology by lactate perfusion

**Figure 2:**
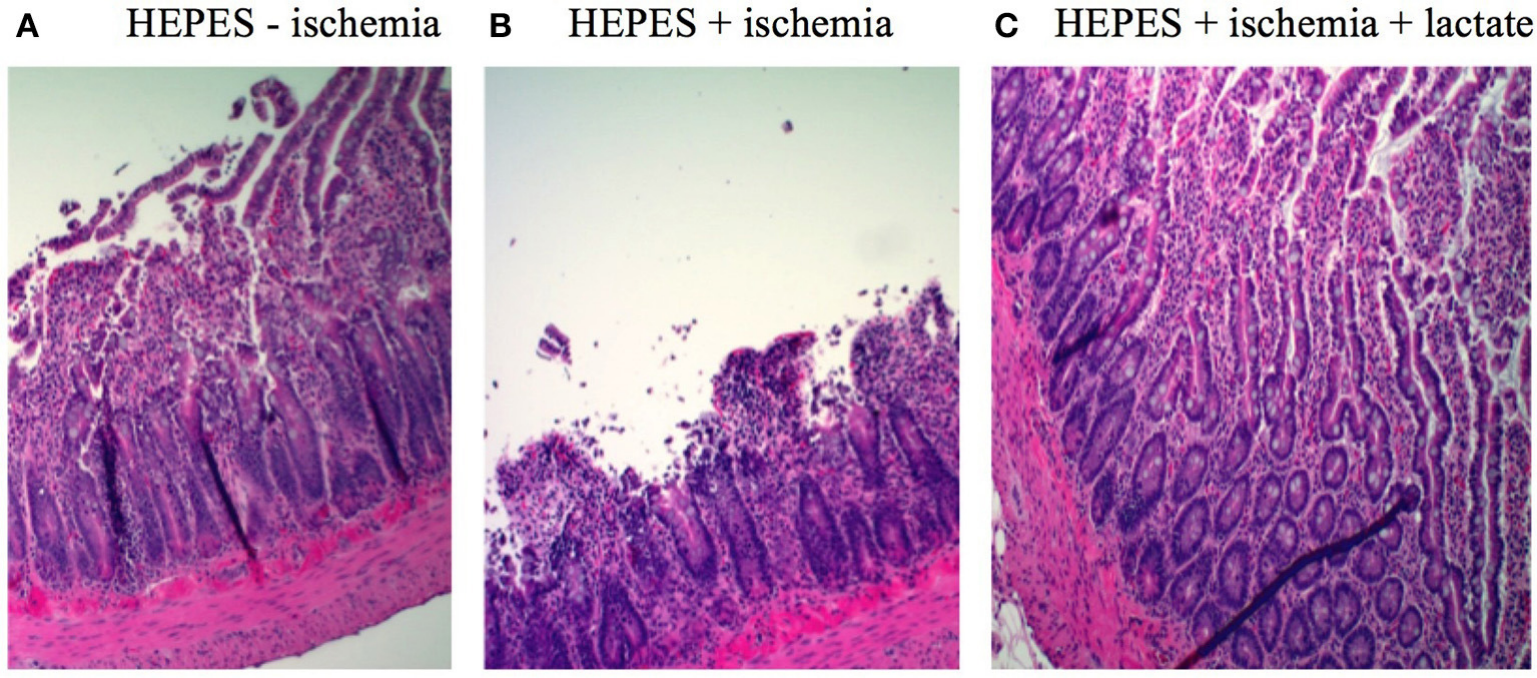
H&E-etched and stained sections of intestinal sections: **(A)** Control without ischemic induction exhibiting sub-epithelial blebbing at the tip of the villus (grade 1 in the Park-Chiu ischemia histopathological classification). **(B)** Ischemic conditions exhibiting in epithelial lifting down to the base of the villi with occasional epithelial breakdown (grade 3). **(C)** Lactate treatment in ischemic conditions exhibiting reversal of pathological abnormalities (grade 1).

After hematoxylin and eosin staining, cross-sections of rat ileum showed an overall normal morphology in control intestines not exposed to ischemia, exhibiting only sub-epithelial blebbing at the tip of the villus (Figure 2A, grade 1 in the Park-Chiu ischemia histopathological classification). Induction of ischemia resulted in epithelial lifting down to the base of the villi with occasional epithelial breakdown (Figure 2B, grade 3). Lactate treatment in ischemic conditions reversed the pathological abnormalities to grade 1 (Figure 2C).

### Lactate's protective effect depends on lactate-to-butyrate conversion

**Figure 3:**
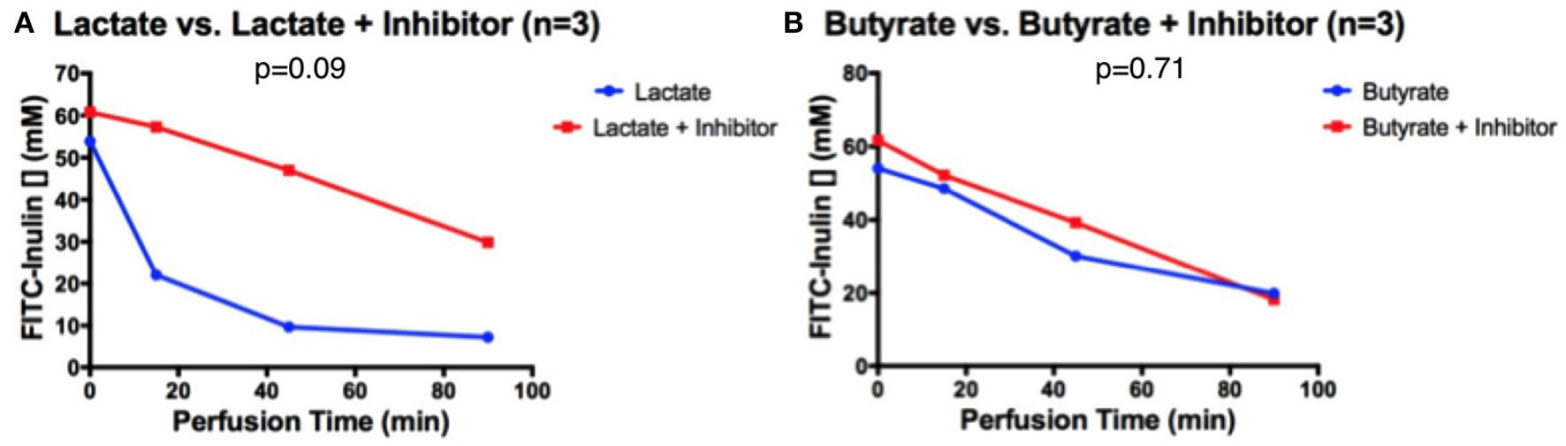
After 30 min of inhibitor pre-treatment, FITC-inulin concentration over 90 min in intestines perfused with HEPES and **(A)** lactate or **(B)** butyrate.

Pre-treatment with an inhibitor of the conversion from lactate to butyrate eliminated the lactate's effect on secretion. In intestines without inhibitor but with lactate, FITC-inulin concentration decreased from 55 to 8 μM after 90 min of perfusion. These exhibited a greater and accelerated decrease in FITC-inulin concentration than intestines with perfused inhibitor and lactate, displaying a final discrepancy of 20 μM at 90 min, although the difference was not statistically significant (*p* = 0.09, Figure 3A). When butyrate was perfused with and without the inhibitor, in both conditions the concentration of FITC-inulin decreased from 60 to 20 μM after 90 min of perfusion; a non-significant difference was observed (*p* = 0.71, Figure 3B). This secretion pattern was similar to that of intestines perfused only with HEPES buffer in ischemic conditions.

### Antibiotic pre-conditioning eliminates lactate's protective effect

**Figure 4:**
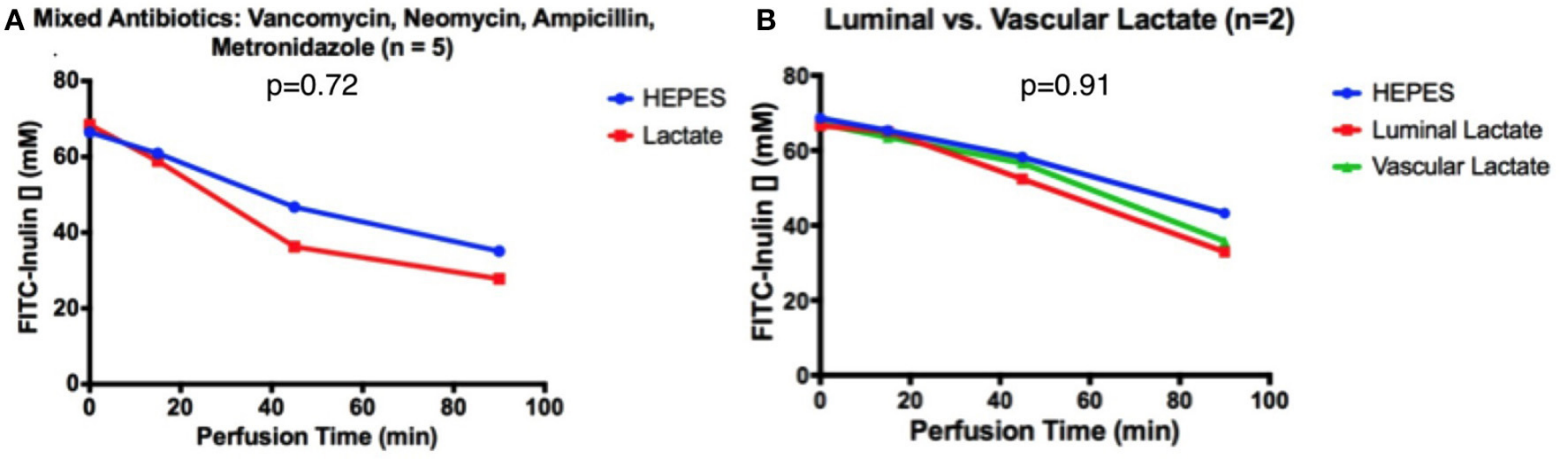
After 30 min of antibiotic pre-treatment, FITC-inulin concentration over 90 min in intestines perfused with HEPES and **(A)** luminal lactate or **(B)** luminal or vascular lactate.

When intestines were pre-treated for 30 min with an antibiotic cocktail (comprised of Vancomycin, Neomycin, Ampicillin, Metronidazole), FITC-inulin concentration decreased from 65 to 35 μM after 90 min of perfusion, both in intestines with and without luminal lactate perfusion; a non-significant difference was observed (*p* = 0.72, Figure 4A). This secretion pattern was similar to that of intestines perfused only with HEPES buffer in ischemic conditions. Similarly, vascular perfusion of lactate did not affect secretion patterns after antibiotic pre-treatment, in fact FITC-inulin concentration decreased from 66 to 33 μM FITC after 90 min of perfusion; a non-significant difference was observed (*p* = 0.91, Figure 4B).

## Discussion

Intestinal IRI causes morbidity and mortality in various intestinal diseases. During intestinal transplantation, the graft is inevitably subjected to ischemic injury. This process alters the integrity of the mucosal enteric barrier, facilitating bacterial translocation and sepsis. The synthesis of pro-inflammatory factors and cytokines contributes to local and systemic inflammatory responses and leads to distant damage to organs such as the liver and lungs, predisposing the patient to multi organ failure and death. The establishment of anti-ischemic methods is a key objective of research in the field of intestinal transplantation. Although numerous techniques have been presented to mitigate IRI, there is as yet, no agreement on the most successful strategy to improve survival and clinical outcome.

In the present study we demonstrate that lactate, a butyrate precursor, has a marked augmenting effect on intestinal epithelial cell secretion, indicating reduced ischemic injury. This effect against intestinal ischemia likely involves ion channels retaining functional activity, even during ischemic insult, thereby allowing greater fluid exchange and resulting in reduced epithelial cell swelling, achieved by greater ion and fluid flow into the lumen. This effect, however, was larger and faster with infused butyrate, suggesting an incomplete conversion of lactate to butyrate.

The pathway of this protective effect is through the conversion of lactate into butyrate-butyrate alone exhibits a protective effect, as does lactate alone. If lactate is perfused along with an inhibitor of conversion of lactate into butyrate, however, the protective effect is lost. Since lactate's protective effect is not observed after antibiotic treatment, the effect appears to be mediated by the microbiome, suggesting microbial involvement in the conversion of lactate to butyrate, thereby generating the protective anti-ischemic effect. The specific commensal flora responsible for this effect are not yet known, and is subject of future study.

Regarding the histopathology, not only lesser ischemic damage but also a difference in length of the villi between the intestinal samples of experimental and control groups was observed. As reported in the literature, butyrate diet supplementation in pigs stimulates villi elongation in both the ileum and the caecum crypts (Yamazoe et al., 2005). Butyrate accelerates epithelial proliferation and increases the crypt cell production rate; it is probably this trophic effect that protects the intestinal barrier during ischemic conditions. Butyrate additionally has considerable antioxidant, anti-inflammatory and anti-apoptotic advantages (Aguilar et al., 2014; Jahns et al., 2015; Pant et al., 2017).

Antibiotic exposure disrupts the microbial community and damages epithelial barrier function (van Ampting et al., 2010). Therefore this study opens a new frontier about the potential correct use of antibiotics in patients experiencing intestinal disease or preparing to undergo intestinal resection or intestinal transplant. It has become a common prophylactic procedure to pretreat the patient with antibiotics prior to the procedure. The results of our study might also affect the current transplant policy of using antibiotic-based decontamination solution during the intestinal procurement and during the initial post-operative period until the enteral feeds are initiated, and would also propose lactate as a potential therapeutic in perioperative care, as lactate's protective effect would likely be amplified if perfused before injury. With these novel findings, the expense and difficulties of direct butyrate administration can be avoided while harnessing butyrate's significant beneficial effects through microbial conversion to reduce ischemic injury and ameliorate intestinal preservation.

## Author contributions

AB contributed to study conception and design, data acquisition, data interpretation, statistical analysis, manuscript drafting. CD contributed to study conception and design, data acquisition, data interpretation, statistical analysis. GC contributed to data acquisition, data interpretation, statistical analysis, manuscript drafting. FD contributed to study conception and design, critical revision. RM contributed to data interpretation, critical revision. MIR and DM contributed to critical revision, funding. MAK contributed to study conception and design, critical reivision, funding. JG contributed to study conception and design, data interpretation, manuscript drafting, critical revision, funding.

### Conflict of interest statement

The authors declare that the research was conducted in the absence of any commercial or financial relationships that could be construed as a potential conflict of interest.

## Appendix

**Table A1:** Experimental conditions corresponding to Figure 4.

	**Lumen**	**Extra-lumen**
Control	HEPES FITC	HEPES bubbled
Exp 1	HEPES FITC	HEPES bubbled + lactate 10 mM
Exp 2	HEPES FITC+ lactate 10 mM	HEPES bubbled

**Table A2:** Park Chiu Classification (Oltean and Olausson, 2010).

**Grade description**	
Grade 0	Normal mucosa
Grade 1	Subepithelial bleb at the tip of the villus
Grade 2	More extended subepithelial space (upper half of the villus)
Grade 3	Epithelial lifting down to the base of the villus, occasional epithelial breakdown
Grade 4	Frequent denuded villi
Grade 5	Loss of villous tissue
Grade 6	Crypt layer destruction
Grade 7	Transmucosal infarction
Grade 8	Transmural infarction

## Abbreviations

IRI: ischemia/reperfusion injury
IBD: inflammatory bowel disease
SCFAs: short-chain fatty acids
IPU: intestinal perfusion unit
H&E: hematoxylin and eosin.

## References

[B1] Aguilar E. C. Leonel A. J. Teixeira L. G. Silva A. R. Silva J. F. Pelaez J. M.. (2014). Butyrate impairs atherogenesis by reducing plaque inflammation and vulnerability and decreasing NFkappaB activation. Nutr. Metab. Cardiovasc. Dis. 24, 606–613. 10.1016/j.numecd.2014.01.002 24602606

[B2] Bourriaud C. Robins R. J. Martin L. Kozlowski F. Tenailleau E. Cherbut C.. (2005). Lactate is mainly fermented to butyrate by human intestinal microfloras but inter-individual variation is evident. J. Appl. Microbiol. 99, 201–212. 10.1111/j.1365-2672.2005.02605.x 15960680

[B3] Duncan S. H. Louis P. Flint H. J. (2004). Lactate-utilizing bacteria, isolated from human feces, that produce butyrate as a major fermentation product. Appl. Environ. Microbiol. 70, 5810–5817. 10.1128/AEM.70.10.5810-5817.2004 15466518 PMC522113

[B4] Eltzschig H. K. Eckle T. (2011). Ischemia and reperfusion–from mechanism to translation. Nat. Med. 17, 1391–1401. 10.1038/nm.2507 22064429 PMC3886192

[B5] Falony G. Vlachou A. Verbrugghe K. De Vuyst L. (2006). Cross-feeding between *Bifidobacterium longum* BB536 and acetate-converting, butyrate-producing colon bacteria during growth on oligofructose. Appl. Environ. Microbiol. 72, 7835–7841. 10.1128/AEM.01296-06 17056678 PMC1694233

[B6] Hill D. A. Artis D. (2010). Intestinal bacteria and the regulation of immune cell homeostasis. Annu. Rev. Immunol. 28, 623–667. 10.1146/annurev-immunol-030409-101330 20192812 PMC5610356

[B7] Jahns F. Wilhelm A. Jablonowski N. Mothes H. Greulich K. O. Glei M. (2015). Butyrate modulates antioxidant enzyme expression in malignant and non-malignant human colon tissues. Mol. Carcinog. 54, 249–260. 10.1002/mc.22102 24677319

[B8] Lee Y. K. Menezes J. S. Umesaki Y. Mazmanian S. K. (2011). Proinflammatory T-cell responses to gut microbiota promote experimental autoimmune encephalomyelitis. Proc. Natl. Acad. Sci. U.S.A. 108(Suppl. 1), 4615–4622. 10.1073/pnas.1000082107 20660719 PMC3063590

[B9] Ley R. E. Bäckhed F. Turnbaugh P. Lozupone C. A. Knight R. D. Gordon J. I. (2005). Obesity alters gut microbial ecology. Proc. Natl. Acad. Sci. U.S.A. 102, 11070–11075. 10.1073/pnas.0504978102 16033867 PMC1176910

[B10] Manichanh C. Rigottier-Gois L. Bonnaud E. Gloux K. Pelletier E. Frangeul L.. (2006). Reduced diversity of faecal microbiota in Crohn's disease revealed by a metagenomic approach. Gut 55, 205–211. 10.1136/gut.2005.073817 16188921 PMC1856500

[B11] Munoz-Abraham A. S. Judeeba S. Alkukhun A. Alfadda T. Patron-Lozano R. Rodriguez-Davalos M. I.. (2015). A new method to measure intestinal secretion using fluorescein isothiocyanate-inulin in small bowel of rats. J. Surg. Res. 197, 225–230. 10.1016/j.jss.2015.02.049 25976849

[B12] Oltean M. Olausson M. (2010). The Chiu/Park scale for grading intestinal ischemia-reperfusion: if it ain't broke don't fix it! Inten. Care Med. 36, 1095. 10.1007/s00134-010-1811-y 20237765

[B13] Pant K. Yadav A. K. Gupta P. Islam R. Saraya A. Venugopal S. K. (2017). Butyrate induces ROS-mediated apoptosis by modulating miR-22/SIRT-1 pathway in hepatic cancer cells. Redox Biol. 12, 340–349. 10.1016/j.redox.2017.03.006 28288414 PMC5350572

[B14] Penders J. Thijs C. van den Brandt P. A. Kummeling I. Snijders B. Stelma F.. (2007). Gut microbiota composition and development of atopic manifestations in infancy: the KOALA Birth Cohort Study. Gut 56, 661–667. 10.1136/gut.2006.100164 17047098 PMC1942165

[B15] Qiao Y. Qian J. Lu Q. Tian Y. Chen Q. Zhang Y. (2015). Protective effects of butyrate on intestinal ischemia-reperfusion injury in rats. J. Surg. Res. 197, 324–330. 10.1016/j.jss.2015.04.031 25976850

[B16] van Ampting M. T. Schonewille A. J. Vink C. Brummer R. J. van der Meer R. Bovee-Oudenhoven I. M. (2010). Damage to the intestinal epithelial barrier by antibiotic pretreatment of salmonella-infected rats is lessened by dietary calcium or tannic acid. J. Nutr. 140, 2167–2172. 10.3945/jn.110.124453 20962149

[B17] Wen L. Ley R. E. Volchkov P. Y. Stranges P. B. Avanesyan L. Stonebraker A. C.. (2008). Innate immunity and intestinal microbiota in the development of Type 1 diabetes. Nature 455, 1109–1113. 10.1038/nature07336 18806780 PMC2574766

[B18] Wu H. J. Ivanov I. I. Darce J. Hattori K. Shima T. Umesaki Y.. (2010). Gut-residing segmented filamentous bacteria drive autoimmune arthritis via T helper 17 cells. Immunity 32, 815–827. 10.1016/j.immuni.2010.06.001 20620945 PMC2904693

[B19] Yamazoe Y. Yamada T. Mitsumori K. (2005). Embryo- and testicular-toxicities of methoxyacetate and the related: a review on possible roles of one-carbon transfer and histone modification. Food Safety 3, 92–107. 10.14252/foodsafetyfscj.2015013

